# Effect of Ripening and In Vitro Digestion on Bioactive Peptides Profile in Ras Cheese and Their Biological Activities

**DOI:** 10.3390/biology12070948

**Published:** 2023-07-02

**Authors:** Ahmed Helal, Alice Cattivelli, Angela Conte, Davide Tagliazucchi

**Affiliations:** 1 Department of Food and Dairy Sciences and Technology, Damanhour University, Damanhour 22516, Egypt; ahmed.helal@damanhour.edu.eg; 2 Department of Life Sciences, University of Modena and Reggio Emilia, Via Amendola, 2—Pad. Besta, 42100 Reggio Emilia, Italy; alice.cattivelli@unimore.it (A.C.); angela.conte@unimore.it (A.C.)

**Keywords:** dairy, high-resolution mass spectrometry, peptidomics, anti-hypertensive, health, protein hydrolysis, lactic acid bacteria

## Abstract

**Simple Summary:**

Milk proteins contain peptides encrypted in their sequence with potential biological activities that can be released after protein hydrolysis as occurs during cheese ripening and gastro-intestinal digestion. The present study was designed to monitor the changes in the bioactive peptides’ profile and biological activities of Ras cheese during ripening (from 30 to 180 days) and in vitro digestion through the application of peptidomics. Results showed that ripening determined an increase in the amount of peptides with angiotensin-converting enzyme (ACE)-inhibitory, dipeptidyl-peptidase-IV-(DPP-IV)-inhibitory and antioxidant activities. The amount of these bioactive peptides was further increased after in vitro digestion. The evolution of bioactive peptides during ripening and in vitro digestion was correlated with the biological activities of cheese. The anti-hypertensive peptides VPP and IPP, with proven in vivo effects, were quantified, and the highest concentrations were found in long-ripened Ras cheese. The presence of bioactive peptides in cheese, especially in the long-ripened one, suggests a possible role of cheese consumption in maintaining human health and in the prevention of cardiovascular diseases and type-2 diabetes. Moreover, the present study allowed the identification of the best ripening time needed for the development of the highest biological activities in Ras cheese after digestion with potential industrial applications.

**Abstract:**

The effect of ripening and in vitro digestion on the biological activities, peptide profiles and release of bioactive peptides in Ras cheese has been investigated. Ras cheese ripening largely influenced the extent of protein hydrolysis. The advancement in ripening resulted in an increase in total peptides (from 0.97 to 2.46 mmol leucine/g in samples at 30 and 180 days of ripening, respectively) and bioactive peptides concentration, especially angiotensin-converting enzyme (ACE)-inhibitory, dipeptidyl-peptidase-IV-(DPP-IV)-inhibitory and antioxidant peptides. In vitro gastro-intestinal digestion further promoted protein hydrolysis and the release of bioactive peptides. Digested Ras cheese at 90 and 180 days of ripening displayed the highest bioactive peptides intensity. The variations in bioactive peptides amount during ripening and in vitro digestion were correlated with the changes in ACE-inhibitory, DPP-IV-inhibitory and antioxidant activities. The highest amounts of VPP and IPP were detected in digested Ras cheese at 90 days of ripening (17.44 and 36.50 mg/kg of cheese, respectively), whereas the highest concentrations of APFPE were found in undigested and digested 180-day ripened Ras cheese (82.09 and 52.01 mg/kg of cheese, respectively). The present investigation underlined potential differences in the biological effect after the ingestion of Ras cheese at different ripening times.

## 1. Introduction

Ras or Roumy cheese is one of the main traditional dairy products in Egypt, and it is widely consumed in Arabic and Mediterranean countries. It is a hard cheese that may be reminiscent of Greek kefalotyri cheese because of its sharp flavour [1,2]. The production of this traditional cheese is limited to small farms located in the Delta region. Ras cheese is typically produced from cow milk or a mixture of cow and buffalo milk without the addition of starter cultures [3,4]. The manufacturing of Ras cheese starts with the addition of rennet to promote milk coagulation followed by heating at 45 °C for 45 min, and then, it is ripened up to 3 months. As a result of the missing addition of starter lactic acid bacteria, the fermentation process of Ras cheese is entirely due to the contaminant microflora (such as lactic acid bacteria, moulds, and yeasts) present in raw milk and/or in the surrounding environment [1].

Ripening is one of the key steps in the cheese production process. During this phase, due to the numerous biochemical and microbiological reactions, the characteristic aroma and texture of the final product is formed [5,6]. Lipolysis, glycolysis and, most importantly, proteolysis are examples of the many biochemical reactions taking place during cheese ripening. Proteolytic activity during cheese manufacturing and ripening is due to the presence of milk endogenous proteolytic enzymes (such as plasmin and somatic cell proteases) as well as of added chymosin and lactic acid bacteria proteases. Initially, caseins are hydrolysed by milk proteolytic enzymes and chymosin to large- and intermediate-size peptides. Next, the formed peptides are further hydrolysed into small peptides or free amino acids by proteinases and peptidases from starter and non-starter lactic acid bacteria as well as from adventitious microflora [5,7,8]. The first step of casein hydrolysis by lactic acid bacteria involves the proteolytic action of cell-envelope proteinases (CEPs) [9]. These enzymes are serine-proteases which are able to cleave caseins into medium-size oligopeptides. CEPs present a broad cutting specificity with a preference for the amino acids Q, N and M at the P1 position and for the amino acids A, G, H and D at the P1’ position [9]. In β-casein, CEPs cleave preferentially the C-terminal and other hydrophobic regions, whereas in αS1-casein, their cleavage preference is located in the N-terminal region [9]. Oligopeptides formed by CEPs action are further transported into the bacterial cells where they are then converted in short peptides and free amino acids by the action of cytoplasmatic peptidase [10]. Lactic acid bacteria synthesise a wide array of cytoplasmatic peptidases which include endopeptidases, aminopeptidases, and di/tri-peptidases with different and complementary specificity [10,11]. The first class of peptidases involved in the hydrolysis of medium-size oligopeptides produced by CEPs includes endopeptidases and aminopeptidases followed by the action of di/tri-peptidases [10,11]. Since milk proteins are particularly rich in proline, it is not surprising that several aminopeptidases and di/tri-peptidases have a marked preference for cleaving peptidic bonds that involve a proline residue [10,11]. Although lactic acid bacteria proteolytic enzymes are responsible for the ripening of almost all cheeses, in some cheese varieties, secondary microflora (such as bacteria from genera *Propionibacterium* and *Brevibacterium* or proteolytic yeast) may have an important role in proteins and oligopeptides hydrolysis, contributing to cheese ripening [12]. 

Previous studies highlighted that proteolysis during cheese manufacturing and ripening promoted the release of peptides with several biological activities [6,7,8,13]. Bioactive peptides are small peptides that exert proven beneficial effects on human health, which are normally encrypted in the parent proteins and released upon hydrolysis. The main sources of bioactive peptides are cow milk, cheese, and dairy products. In addition, they can also be found in other animal products such as meat, eggs, fish, tuna, herring, and salmon, and plant-based products such as corn, rice, and mushrooms [13]. Bioactive peptides normally range in length from 2 to 20 amino acids and, depending on their composition and amino acid sequence, exert different bioactivities [6]. Various physiological functions have been ascribed to bioactive peptides, among others antioxidant, antibacterial, immunomodulatory, cholesterol-lowering, and opioid activities [7]. In addition, bioactive peptides can inhibit the action of some enzymes involved in the onset of chronic diseases such as dipeptidyl peptidase-IV (DPP-IV) and angiotensin-converting enzyme (ACE). The inhibition of these enzymes is crucial for the prevention of cardiovascular diseases and diabetes pathogenesis.

Dipeptidyl peptidase-IV is an intestinal membrane-bound peptidase whose function is to hydrolyse and inactivate the insulinotropic hormones incretins. The inhibition of DPP-IV may prolong the half-life of incretins and therefore indirectly of insulin, achieving a reduction in hyperglycaemia [14].

Angiotensin-converting enzyme (ACE) is involved in the conversion of the plasma peptide angiotensin I to angiotensin II. Angiotensin II possesses strong vasoconstrictor power and for this reason leads to an increase in blood pressure. ACE inhibition is fundamental to blood pressure regulation; indeed, ACE is the target of the pharmacological treatment of hypertension and related cardiovascular diseases [15].

The identification and quantification of bioactive peptides directly in cheese (or in general in foodstuff) is not enough for the prediction of potential in vivo effects, as the peptides’ structure may be thoroughly modified during digestion. Therefore, the beneficial effects of bioactive peptides on human health depend on their stability during gastro-intestinal digestion. During digestion, in fact, bioactive peptides can be degraded or transformed into new bioactive sequences by the action of gastric and intestinal enzymes such as pepsin and pancreatic proteases [8]. Numerous studies have shown that in vitro digestion greatly affected the peptide profile of cheese with some peptides that were degraded during digestion, while others were released or increased in concentration [7,16,17]. 

Therefore, the aim of this work was to investigate the effect of ripening and in vitro gastro-intestinal digestion on Ras cheese to evaluate the evolution and fate of bioactive peptides and related biological activities. High-resolution mass spectrometry coupled with in silico peptidomics analysis was carried out to elucidate semi-quantitative changes in the bioactive peptides amount as affected by ripening and in vitro digestion. Some bioactive peptides with proven in vivo activity were also absolutely quantified.

## 2. Materials and Methods

### 2.1. Materials

Chemicals for high-resolution mass spectrometry analysis were purchased from Biorad (Hercules, CA, USA), whereas all the other chemicals and reagents were supplied by Sigma-Aldrich (Milan, Italy). Filters for ultrafiltration with a cut-off of 3 kDa were supplied by Millipore (Milan, Italy). The standard for peptides quantification (APFPE, IPP and VPP; >99% purity) was purchased from Bio-Fab Research (Rome, Italy). Raw cow milk was supplied by a local animal farm (Al Nubaria, Egypt). Annatto 055-MF-WS, natural water-soluble food grade colour, was purchased from MIFAD (Cairo, Egypt), whereas rennet (RENIPLUS^®^ dried microbial rennet from plant origin) was from Proquiga Biotech (La Coruña, Spain). 

### 2.2. Ras Cheese Manufacturing and Compositional Analysis

Ras or Roumy cheese was manufactured following the method described in Hofi et al. [18] with some modifications. Firstly, raw cow milk was mixed with annatto and warmed to 32 °C, after which rennet was added. After 45 min of incubation, the obtained curd was cut vertically and horizontally into small cubes using stainless-steel knives. Then, the temperature was increased to 45 °C in 15 min and the curd was cooked for 30 min under gentle stirring until the whey acidity reached 0.14%. After that, one-third of cheese whey was drained off, 4% NaCl was added, and the curd was left at 45 °C for an additional 15 min. After draining the remaining whey, the curd was cooled, moulded, and pressed overnight. Cheese salting was applied by using ground salt sprinkled to the cheese surface. Cheese was then stored for 30, 90 and 180 days at 13 ± 2 °C and 80 ± 5% relative humidity. Three different batches of Ras cheese were prepared at the different ripening time for the next analysis.

The total protein content of cheese samples was determined by measuring the total nitrogen content using the Kjeldahl method [19] and expressed as total protein in dry matter. The water-soluble protein content was measured as described by Kuchroo and Fox [20]. The soluble protein coefficient was calculated as the percentage of water-soluble protein to total proteins. The fat content was measured with the Gerber method, whereas the NaCl content was determined with the Volhard method [19,21]. Analyses have been carried out in triplicate.

The gross chemical composition of the Ras cheeses is reported in Supplementary Table S1.

### 2.3. Extraction of Water-Soluble Peptides (WSPs) from Ras Cheese

The extraction of WSPs from Ras cheeses was conducted as described in Martini et al. [14]. A volume of 45 mL of HCl 0.1 mol/L was added to 5 g of cheese, and the sample was subsequently homogenised with an Ultra-Turrax homogeniser. A total of 3 cycles of 1 min of homogenisation, interspersed with 1 min of resting on ice, were carried out. The obtained homogenate was then centrifuged at 4000× *g*, 4 °C for 40 min before filtering with Whatman filter paper 4. WSP extraction was carried out in triplicate, and the replicates were pooled together before analysis.

### 2.4. In Vitro Gastro-Intestinal Digestion of Ras Cheeses

Ras cheeses at different ripening times were subjected to in vitro gastro-intestinal digestion following the INFOGEST protocol as described by Brodkorp et al. [22]. For the oral phase of the digestion, 1 g of Ras cheese was mixed with 1 mL of simulated salivary fluid, to which we added human salivary α-amylase (150 U/mL). After homogenisation, the bolus was then incubated for 2 min at 37 °C in a rotating wheel (10 rpm). Next, 2 mL of gastric fluid and pepsin (final concentration in the digestive system of 2000 U/mL) was added to the bolus, and the pH was brought to 3 with 6 mol/L HCl. The gastric phase was simulated by incubating the bolus at 37 °C for 120 min under rotation (10 rpm). At the end of the gastric phase, the chyme was mixed with 4 mL of intestinal fluid, the pH was raised to 7.5 and the chyme was incubated at 37 °C for 30 min under rotation (10 rpm) before adding pancreatin (final concentration based on trypsin activity of 200 U/mL). To simulate the intestinal phase, the chyme was then incubated for 120 min at 37 °C under rotation (10 rpm). Samples withdrawn at the end of the digestion were then incubated for 5 min in boiling water to inactivate the proteases following centrifugation at 10,000× *g*, 4 °C for 20 min.

A control digestion in which cheese was replaced by water was carried out to consider the possible interferences of the digestive system in the applied assays.

Digestions were carried out in triplicate for each sample, and the replicates were pooled together before the analysis. 

The full protocol with the description of the different steps and the digestive fluids composition can be found in Brodkorp et al. [22].

### 2.5. Peptides Quantification and Analysis of the Biological Activities in Undigested and Digested Ras Cheeses

The total amount of peptides in WSP fractions and in in vitro digested samples was determined as free amino groups by applying the TNBS method described in Adler-Nissen [23]. The data are presented as mmol of leucine equivalents/g of cheese and corrected for the contribution of the control digestion.

The analysis of the biological activities was performed on low molecular weight peptide fractions (LMWPFs) extracted from WSP and from in vitro digested samples by ultrafiltration at 3 kDa cut-off, as previously reported [15]. 

The antioxidant activity of LMWPFs was determined by two different assays. The ABTS (2,2-azino-bis(3-ethylbenzothiazoline-6-sulphonic acid)) assay was carried out as described in Re et al. [24], and the results were reported as mg of ascorbic acid/100 g of cheese. On the other hand, a FRAP (ferric-reducing antioxidant power) assay was performed following the protocol reported in Benzie and Strain [25]. Results were expressed as mg of ascorbic acid/100 g of cheese.

The angiotensin-converting enzyme (ACE) inhibitory activity of LMWPF was determined by applying the method reported in Ronca-Testoni adapted to a microplate reader [26,27]. The used substrate was the tripeptide N-[3-(2-furyl)acryloyl]-L-phenylalanylglycyl-glycine (FAPGG), and the results were expressed as IC_50_ values, which are defined as the amount of cheese (mg of cheese/mL) able to inhibit by 50% the enzymatic activity. The IC_50_ values were determined as described in Helal et al. [28].

The ability of the LMWPF to inhibit the enzyme dipeptidyl-peptidase-IV (DPP-IV) was assessed by using the peptide glycine-proline-*p*-nitroanilide as substrate and following the protocol described in Tagliazucchi et al. [29]. Data are reported as IC_50_ values calculated as previously described [28].

### 2.6. Peptidomics Analysis and Label-Free MS Peak Quantification

LMWPFs extracted from WSP and from in vitro digested Ras cheese samples were subjected to high-resolution mass spectrometry analysis to establish the peptide profiles of the different samples. The instrument used was a Q Exactive Hybrid Quadrupole-Orbitrap Mass Spectrometer (Thermo Scientific, San Jose, CA, USA) coupled with a UHPLC system (UHPLC Ultimate 3000 separation module, Thermo Scientific, San Jose, CA, USA). Peptide separation was achieved by using a C18 column (Acquity UPLC HSS C18 reversed phase, 2.1 × 100 mm, 1.8 µm particle size, Waters, Milan, Italy). The chromatographic conditions, the mass spectrometry and tandem mass spectrometry parameters are fully described in Martini et al. [14]. 

For peptide identification, the raw files from high-resolution mass spectrometry analysis were converted in Mascot generic files (.mgf), and peptide identification was performed through the Mascot software as reported in Martini et al. [14]. Peptide identification was considered successful with a sequencing significance threshold of *p* < 0.05. 

The list of peptides identified by Mascot software in the different samples was converted in .dat files and used for label-free MS peak quantification through the Skyline software (Skyline v21.2) [30] and applying the protocol developed by Dallas and Nielsen [31]. 

Initially, a specific peptide library was created in Skyline software by pooling together the lists of peptides identified by Mascot software in the different undigested and digested Ras cheese samples. Next, the raw mass spectral data obtained by high-resolution mass spectrometry from the different samples were processed for the MS peak quantification of each identified peptide [31]. Skyline software was developed to find peptides in a specific sample by matching the retention time and the *m/z* value with the peptides originally identified with Mascot software. This means that Skyline software search for specific peptides present in the created library in all the analysed samples, whether or not they have been previously sequenced and identified by Mascot software, by comparing the retention time and the *m/z* value. The accuracy of the identification in Skyline was expressed as odt value. As suggested by the developer of the Skyline software, only peptides with odt ≥ 0.8 were considered significant and correctly identified. Indeed, all the *m/z* signals with an error > of ±5 ppm were not considered.

Only peptides belonging to the major milk proteins (β-casein, αS1-casein, αS2-casein, κ-casein, β-lactoglobulin and α-lactalbumin) were included in the following analysis.

### 2.7. Bioactive Peptides Identification and Semi-Quantitative Analysis, and Absolute Quantification of the Bioactive Peptides VPP, IPP and APFPE

The search for the presence of peptides with previously reported biological activity (100% sequence homology) was performed by using the Milk Bioactive Peptide Database (MBPDB, http://mbpdb.nws.oregonstate.edu/ accessed on 6 April 2023) [32]. A semi-quantitative analysis of identified bioactive peptides was carried out through the Skyline dataset of MS quantitative data. The peak area values of the same peptide but with different modifications (such as different protonation pattern, methionine oxidation and glutamine/asparagine deamidation) were summed.

Absolute quantification of the bioactive peptides VPP, IPP and APFPE was carried out by applying the parallel reaction monitoring method developed by Martini et al. [7]. The calibration curves were built by using synthetic peptides with a purity ≥99%.

### 2.8. Statistical Analysis

All the data were expressed as mean ± standard deviation (SD) for triplicate analytical replications for each sample. One-way ANOVA and Tukey a post hoc test were applied for statistical analysis (GraphPad Prism 6.0 Software, San Diego, CA, USA). Differences were considered significant when *p* < 0.05.

## 3. Results and Discussion

### 3.1. Peptide Quantification and Biological Activities of Undigested and Digested Ras Cheeses at Different Ripening Times

**Figure 1:**
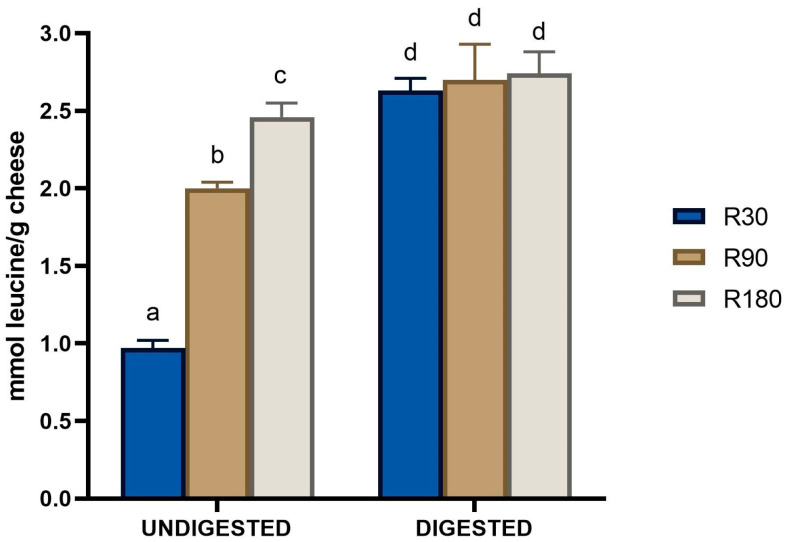
Variation in protein hydrolysis of Ras cheeses at different ripening times before and after in vitro gastro-intestinal digestion. Analysis was performed on the water-soluble peptide fractions extracted from undigested Ras cheese samples as well as on the in vitro digested Ras cheese samples. R30: Ras cheese at 30 days of ripening; R90: Ras cheese at 90 days of ripening; R180: Ras cheese at 180 days of ripening. Proteolysis was determined as concentration of free amino groups by using the TNBS assay. The data are expressed as mmol of leucine equivalent/g of cheese. The in vitro digestion data were corrected for the contribution of the control digestion. Values are means of three assay replications ± standard deviation (SD). Different letters among samples denote significant differences (*p* < 0.05).

As expected, Ras cheese ripening resulted in a significant increase in the amount of water-soluble peptides (WSP), which were quantified as free amino groups (Figure 1).

The total WSP concentration in 30-days ripened Ras cheese was 0.97 ± 0.05 mmol leucine equivalent/g, and their amount almost doubled after 90 days of ripening (2.00 ± 0.04 mmol leucine equivalent/g). Next, the total amount of WSP further increased significantly after 180 days of ripening, reaching a value of 2.46 ± 0.09 mmol leucine equivalent/g. In agreement, previous studies found a ripening-dependent increase in the amount of released peptides in different types of cheese [6,7,16,33]. Kocak et al. [34] found that the amount of released amino groups increased during the ripening of white-brined cheese and almost duplicate passing from 30 to 90 days of ripening. Similar results were also obtained by Baptista et al. [35] with Prato cheese and by Galli et al. [36] with Asiago cheese. However, long ripening (i.e., more than 12 months) determined the achievement of a plateau in the released peptides as observed for Grana Padano and Parmigiano Reggiano cheeses [7,37].

In vitro digestion of Ras cheeses brought about a significant increase in the amount of released peptides in all the samples (Figure 1). The highest increase of 2.71 times was observed in 30-days ripened Ras cheese, whereas the lowest one (1.11 times) was obtained in digested Ras cheese at 180 days of ripening. After digestion, no significant differences in the amount of peptides were found among the Ras cheese samples. To the best of our knowledge, only one study addressed the combined effect of ripening and in vitro digestion on the total amount of peptides released from cheese. In addition, in this study, the in vitro digestion of Parmigiano Reggiano cheese brought about an increase in the concentration of peptide, with respect to the undigested cheese samples, without significant differences as a function of the ripening time. 

**Figure 2:**
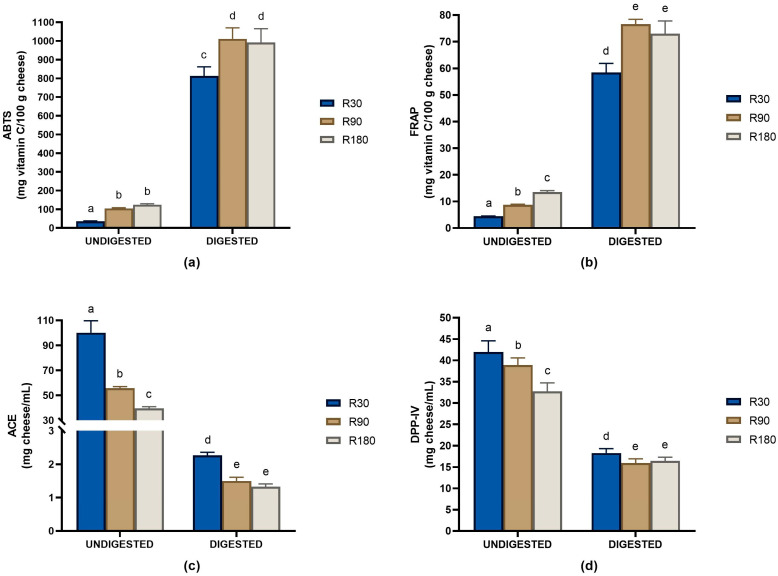
Biological activity profiles of Ras cheeses at different ripening times before and after in vitro gastro-intestinal digestion. Biological activities analysis was carried out on the low molecular weight peptide fractions (<3 kDa) obtained from the water-soluble peptide fractions of undigested Ras cheese and from in vitro digested Ras cheese samples. R30: Ras cheese at 30 days of ripening; R90: Ras cheese at 90 days of ripening; R180: Ras cheese at 180 days of ripening. Antioxidant activity was assessed with the ABTS (**a**) and FRAP (**b**) assays. ACE-inhibitory activity (**c**) and DPP-IV-inhibitory activity (**d**) were reported as IC_50_ values defined as the cheese amount, expressed in mg of cheese/mL, able to inhibit by 50% the enzyme activity. The in vitro digestion data were corrected for the contribution of the control digestion. Values are means of three assay replications ± standard deviation (SD). Different letters among samples in the same assay denote significant differences (*p* < 0.05).

Both ripening and in vitro digestion also affected the biological activity profiles of Ras cheese (Figure 2).

In general, ripening time improved the biological properties of Ras cheese as evidenced by the increase in antioxidant activity and the decrease in the IC_50_ values against ACE and DPP-IV during ripening. In vitro digestion further strengthens the bioactive features of Ras cheese. Antioxidant activity determined with both the ABTS and FRAP assays strongly increased after in vitro digestion and displayed a ripening time dependence (Figure 2a,b). Antioxidant activity significantly increased passing from in vitro digested Ras cheese at 30 days of ripening to in vitro digested Ras cheese at 90 days of ripening to then plateau. No significant differences were found in the antioxidant activity determined with both the ABTS and FRAP assays between in vitro digested Ras cheeses at 90 and 180 days of ripening. Regarding the ACE-inhibitory activity, a decrease between 30 and 44 times in the IC_50_ values after in vitro digestion was observed, which indicated an increase in the ACE-inhibitory activity in digested Ras cheeses (Figure 2c). The ACE-inhibitory activity increased during ripening, and Ras cheese at 30 days of ripening displayed significantly higher IC_50_ value with respect to Ras cheeses at longer ripening time. Among the digested samples, the lower ACE-inhibitory activity was observed in in vitro digested Ras cheese at 30 days of ripening, whereas no significant differences were found between in vitro digested Ras cheeses at 90 and 180 days of ripening. In addition, IC_50_ values against DPP-IV decreased during ripening and further decreased after in vitro digestion. Once again, no significant differences were detected between the Ras cheeses at 90 and 180 times of ripening after in vitro digestion (Figure 2d).

The effect of ripening on the biological activity of cheese has been already reported. For example, Ong and Shah [38] and Baptista et al. [39] recorded an increase in ACE-inhibitory activity during the ripening of Cheddar and Prato cheeses, respectively. On the other hand, Kocak et al. [34] demonstrated that the ripening of goat cheese resulted in an increase both in the antioxidant and ACE-inhibitory activities.

### 3.2. Peptide Profile Evolution during Ras Cheese Ripening

The changes in the peptide profiles of Ras cheeses were followed during ripening both from a qualitative and semi-quantitative point of view. The complete list of the identified peptides with mass spectrometry and semi-quantitative data is reported in Supplementary Table S2.

**Figure 3:**
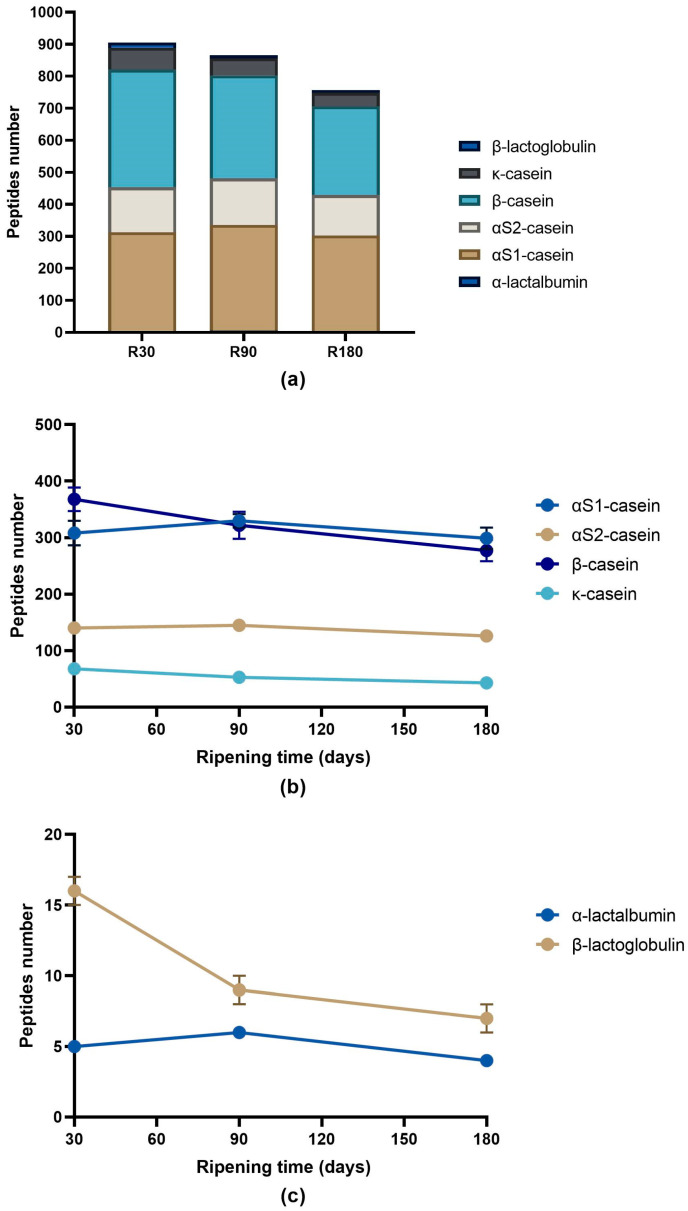
Number of peptides per protein in Ras cheeses at different ripening times. Analysis was carried out on low-molecular weight peptide fractions obtained by ultrafiltration at 3 kDa from the water-soluble peptide fractions extracted from Ras cheese samples at 30 (R30 sample), 90 (R90 sample) and 180 (R180 sample) days of ripening. (**a**) Number of peptides identified in Ras samples released from caseins and whey proteins. (**b**) Evolution of caseins peptide number during Ras cheese ripening. (**c**) Evolution of whey proteins peptide number during Ras cheese ripening. The complete list of identified peptides can be found in Supplementary Table S2.

The number of identified peptides gradually decreased according to the ripening time passing from 905 peptides identified in Ras cheese at 30 days of ripening to 756 peptides identified in Ras cheese at 180 days of ripening (Figure 3a). The majority of the identified peptides in all the samples came from the hydrolysis of β-casein and αS1-casein (74.7%, 75.4% and 76.2% in Ras cheese at 30, 90 and 180 days of ripening, respectively) followed by αS2-casein and κ-casein. Very few identified peptides were derived from the hydrolysis of whey proteins (β-lactoglobulin and α-lactalbumin).

The analysis of the evolution during ripening of the identified peptides grouped by proteins revealed a different trend depending on the protein. The number of peptides released from β-casein constantly decreased during ripening. The same trend was observed for κ-casein- and β-lactoglobulin-derived peptides (Figure 3b,c). Differently, the number of peptides identified from the hydrolysis of αS1-casein, αS2-casein and α-lactalbumin slightly increased from 30 to 90 days of ripening to then drop after 180 days of ripening (Figure 3b,c).

**Figure 4:**
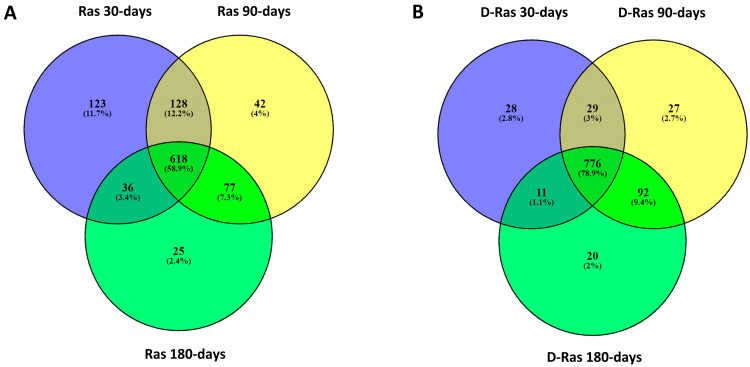
Venn diagrams showing differences in peptide profiles of Ras cheese samples. (**A**) Venn diagram of undigested Ras cheeses. (**B**) Venn diagram of in vitro digested Ras cheeses.

A total of 618 peptides were found in common in the three ripened Ras cheese samples (Figure 4A), suggesting that these peptides are easily released and conserved during ripening. On the contrary, 293 peptides were present in Ras cheese at 30 and 90 days of ripening but absent in the sample at 180 days of ripening. These peptides were probably further hydrolysed by lactic acid bacteria extracellular or intracellular proteases or assimilated by the lactic acid bacteria themselves. Several strains of lactic acid bacteria able to colonise cheese during ripening such as *Lacticaseibacillus casei*, *Lacticaseibacillus rhamnosus*, *Lactobacillus delbrueckii* and others possess a complex proteolytic system which is responsible for the hydrolysis of milk proteins [10,11]. Caseins are firstly hydrolysed by cell envelope proteases in oligopeptides of different lengths that are then internalised into the bacterial cells by peptide transporters and further cleaved into small peptides and free amino acids [10,11].

**Figure 5:**
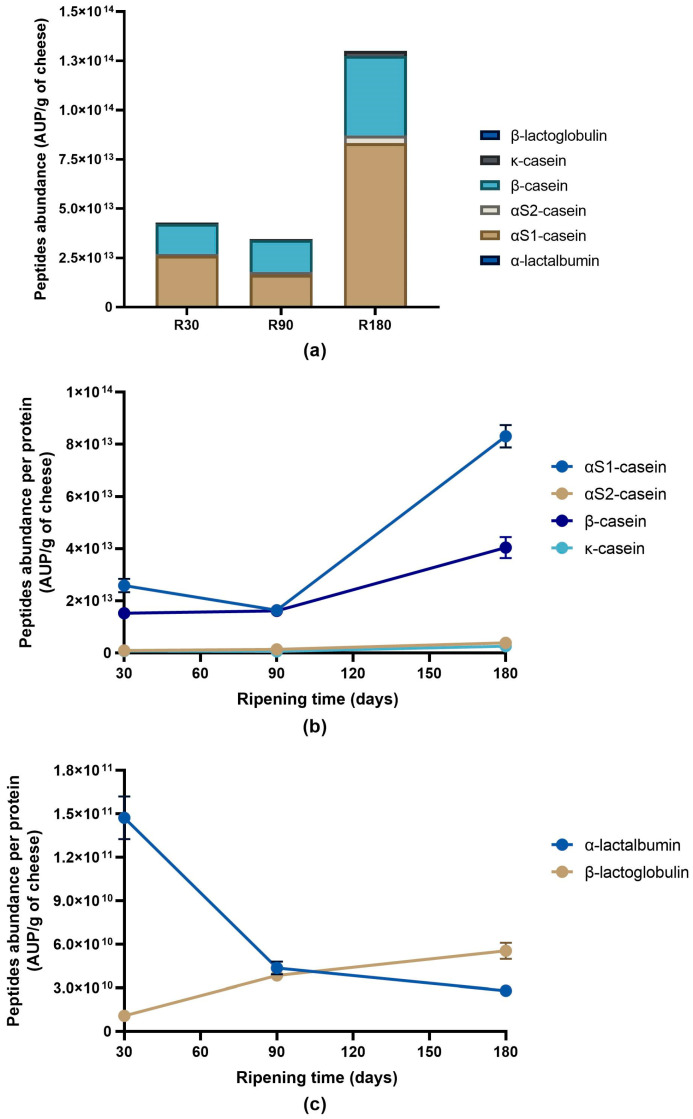
Peptide abundance per protein in Ras cheese at different ripening times. Analysis was carried out on low-molecular weight peptide obtained by ultrafiltration at 3 kDa from the water-soluble peptide fractions extracted from Ras cheese samples at 30 (R30 sample), 90 (R90 sample) and 180 (R180 sample) days of ripening. (**a**) Total peptides abundance per proteins in Ras samples. (**b**) Evolution of caseins peptide abundance during Ras cheese ripening. (**c**) Evolution of whey proteins peptide abundance during Ras cheese ripening. Data are reported as the sum of the intensity of each identified peptide measured as area under the peak (AUP) by Skyline analysis. The complete list of identified peptides and the semi-quantitative data can be found in Supplementary Table S2.

Despite the decreasing trend in peptide number accordingly with the ripening time, semi-quantitative analysis revealed a different tendency among the sum of the intensity (peptide abundance measured as area under the peak for each specific peptide) of the identified peptides in the Ras cheese samples (Figure 5a). 

The peptide abundance displayed a slight decrease passing from Ras cheese at 30 days of ripening to Ras cheese at 90 days of ripening, whereas a 3.8 times increase was detected passing from 90 to 180 days of ripening. Looking at the peptide abundance by protein (Figure 5b,c), αS1-casein and β-casein exhibited the highest peptide abundance in any sample, and αS1-casein overcame β-casein in peptide abundance at each ripening time. The behaviour was quite similar for both the proteins with little or no decrease from 30 to 90 days of ripening and an evident increase in peptide abundance passing from 90 to 180 days of ripening. A similar trend was also observed for κ-casein, whereas the peptide abundance of αS2-casein and β-lactoglobulin continuously increased during ripening. Finally, in the case of α-lactalbumin, the peptides abundance was maximum after 30 days to then decrease after 90 and 180 days of ripening. 

The Intensity of individual peptides identified in the different proteins significantly changed during ripening. In αS1-casein, some peptides were easily released after 30 days of ripening and decreased as the ripening proceeded, whereas other peptides continuously increased in intensity during ripening (Supplementary Table S2 and Figure S1). This was the case of peptides released from the hydrophobic regions 1–36 and 80–115 of αS1-casein. Peptides from these regions were already present after 30 days of ripening, and their intensity had risen during ripening, suggesting that these peptides were constantly released from the parent proteins. The liberation of these peptides was a consequence of the lactic acid bacteria cell-envelope proteinases activity. Previously published papers have suggested that these hydrophobic regions in αS1-casein are more susceptible to hydrolysis by lactic acid bacteria cell-envelope proteinases and contained the preferred cleavage sites of cell-envelope proteinases [9,40]. Differently, peptides in the regions 37–80 and 150–199 were either absent or present in low abundance after 30 days of ripening, but their intensity increased at late ripening. These regions in αS1-casein, therefore, contain secondary cleavage sites which are less efficiently hydrolysed by the cell-envelope proteinases [9,40]. Finally, most peptides from the regions 8–27 and 115–150 displayed a decreasing trend in intensity during ripening, suggesting that they were released at the early stage of ripening and further hydrolysed by lactic acid bacteria proteinases and peptidases.

In the case of β-casein, the majority of peptides from the C-terminal region (between the positions 115 and 209) were promptly released at the beginning of ripening, and their intensity strongly decreased as the ripening proceeded (Supplementary Table S2 and Figure S2). It is well known that most of the characterised lactic acid bacteria cell-envelope proteinases have an established preference for hydrolysing the C-terminal region of β-casein [9,40,41,42]. Anyway, the majority of β-casein-derived peptides displayed an increasing trend in their abundance during ripening. 

### 3.3. Peptide Profile Evolution during Ras Cheese In Vitro Digestion

The in vitro digestion of Ras cheeses at different ripening stages thoroughly modified the peptide profiles of the cheeses (Supplementary Table S2).

**Figure 6:**
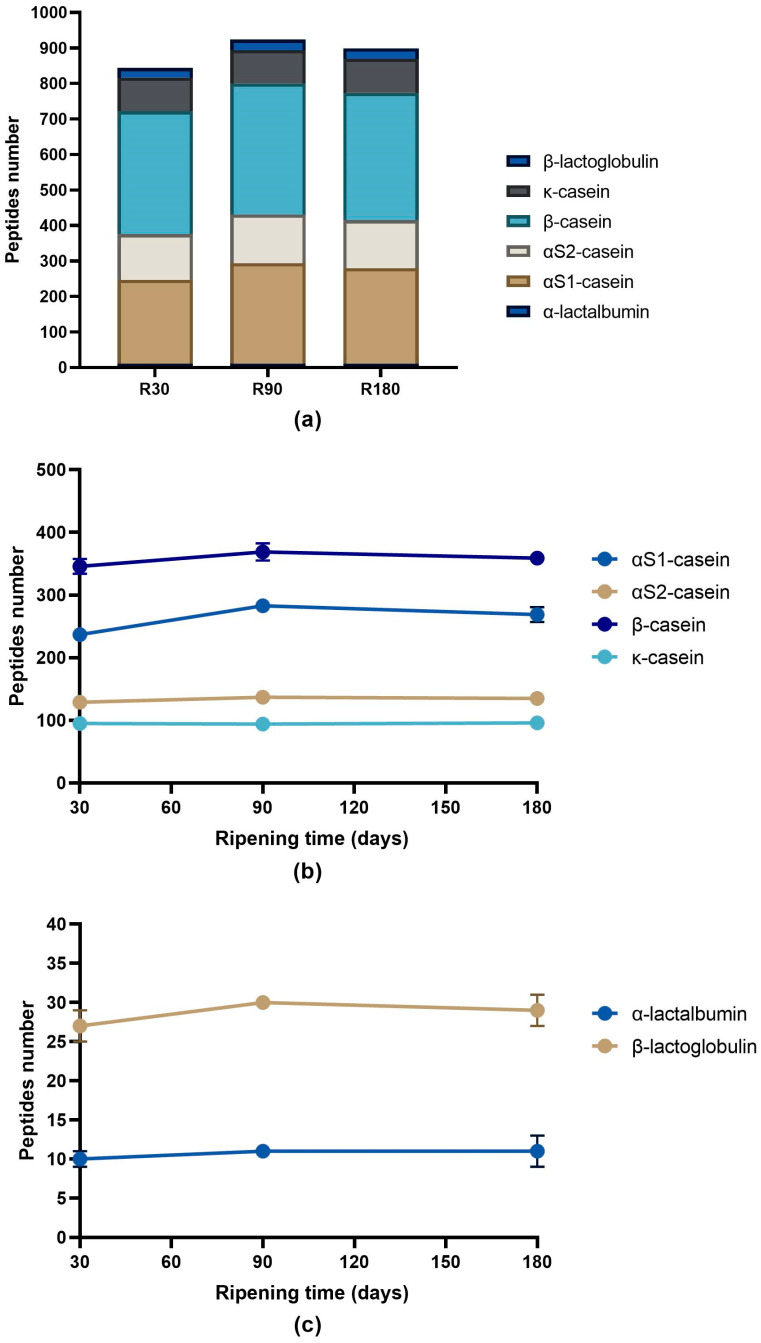
Number of peptides per protein in in vitro digested Ras cheese at different ripening times. Analysis was carried out on low-molecular weight peptide fractions extracted by ultrafiltration at 3 kDa from in vitro digested Ras cheese samples at 30 (R30 sample), 90 (R90 sample) and 180 (R180 sample) days of ripening. (**a**) Number of peptides identified in in vitro digested Ras samples released from caseins and whey proteins. (**b**) Evolution of caseins peptide number during Ras cheese ripening after in vitro digestion. (**c**) Evolution of whey proteins peptide number during Ras cheese ripening after in vitro digestion. The complete list of identified peptides can be found in Supplementary Table S2.

The lowest number of peptides was found for the in vitro digested Ras cheese at 30 days of ripening, whereas the number of identified peptides in in vitro digested Ras cheese at 90 and 180 days of ripening was quite similar (Figure 6a). The majority of identified peptides were from β-casein followed by αS1-casein. Once again, very few peptides were released from whey proteins hydrolysis. The number of identified peptides from β-casein, αS1-casein and β-lactoglobulin increased from in vitro digested Ras cheese at 30 days of ripening to in vitro digested Ras cheese at 90 days of ripening and then reached a plateau (Figure 6b,c). In the case of the other proteins, the number of peptides was similar among the in vitro digested samples at the different ripening times (Figure 6b,c). The Venn diagram revealed that most of the identified peptides (776 peptides corresponding to the 78.9% of the identified peptides in in vitro digested samples) were in common among the different in vitro digested samples (Figure 4B). Therefore, gastric and intestinal proteases that present a broad substrate-specificity tended to level the differences among samples displaying similar peptide profiles. Furthermore, 51.2%, 52.1% and 42.9% of the peptides were conserved during in vitro digestion of Ras cheese at 30, 90 and 180 days of ripening, respectively, since they were already present in the corresponding undigested sample (Supplementary Figure S3).

**Figure 7:**
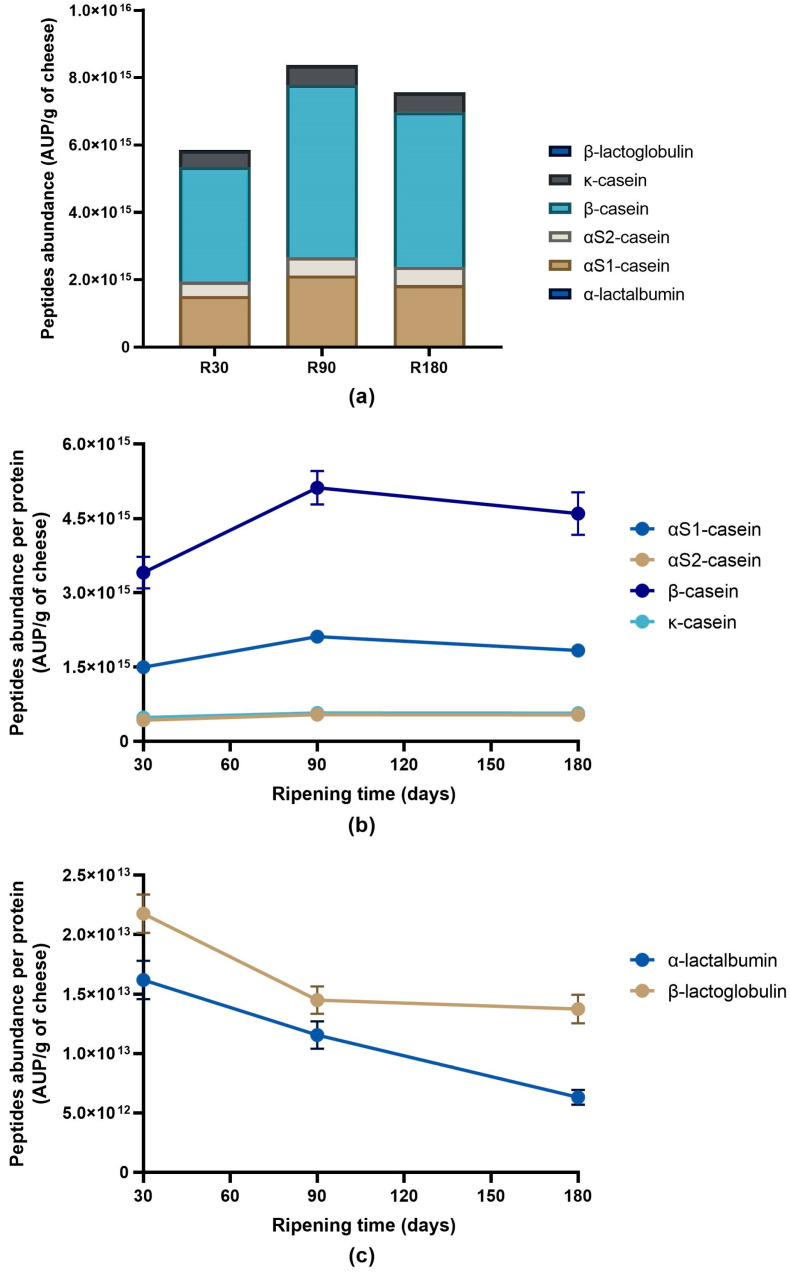
Peptide abundance per protein in in vitro digested Ras cheese at different ripening times. Analysis was carried out on low-molecular weight peptide fractions extracted by ultrafiltration at 3 kDa from in vitro digested Ras cheese samples at 30 (R30 sample), 90 (R90 sample) and 180 (R180 sample) days of ripening. (**a**) Total peptides abundance per proteins in in vitro digested Ras samples. (**b**) Evolution of caseins peptide abundance during Ras cheese ripening after in vitro digestion. (**c**) Evolution of whey proteins peptide abundance during Ras cheese ripening after in vitro digestion. Data are reported as the sum of the intensity of each identified peptide measured as area under the peak (AUP) by Skyline analysis. The complete list of identified peptides and the semi-quantitative data can be found in Supplementary Table S2.

A general increase in total peptide abundance and peptide abundance per protein was observed after in vitro digestion (Figure 7a–c). 

Differently from undigested samples, β-casein was the protein with the highest peptide abundance in any digested sample, which was followed by αS1-casein. The lowest peptide abundance was found for whey proteins. The peptide abundance of β-casein and αS1-casein increased, passing from in vitro digested Ras cheese at 30 days of ripening to in vitro digested Ras cheese at 90 days of ripening to then experience a slight decline in in vitro digested Ras cheese at 180 days of ripening. Similarly, αS2-casein and κ-casein peptide abundance increased, passing from digested Ras cheese at 30 days of ripening to Ras cheese at 90 days of ripening to then reach a plateau. Vice versa, the peptide abundance of whey proteins continuously decreased in digested samples according to the increase in ripening time. With only a few exceptions, the peptides conserved across the digestion were found in higher abundance in digested samples.

### 3.4. Identification of Bioactive Peptide in Ras Cheese Samples before and after In Vitro Digestion

Considering all the samples, a total of 126 peptides with previously reported biological activity were identified. The complete list of detected bioactive peptides together with the alleged bioactivities and the sample in which they were found is reported in Supplementary Table S3.

**Figure 8:**
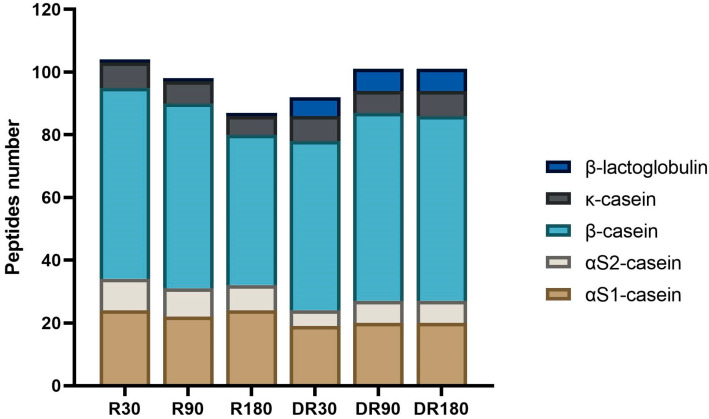
Number of bioactive peptides per protein in undigested and in vitro digested Ras cheese at different ripening times. Analysis was carried out on low-molecular weight peptide fractions extracted by ultrafiltration at 3 kDa from Ras cheese samples at 30 (R30 sample), 90 (R90 sample) and 180 (R180 sample) days of ripening and from in vitro digested Ras cheese samples at 30 (DR30 sample), 90 (DR90 sample) and 180 (DR180 sample) days of ripening. The complete list of identified peptides can be found in Supplementary Table S3.

The highest number of bioactive peptides was found in Ras cheese at 30 days of ripening (104 bioactive peptides) (Figure 8). In general, ripening time had a negative effect on the number of identified bioactive peptides, since their number constantly decreased according to the increase in ripening time.

The effect of the digestion on the number of bioactive peptides was related to the ripening time (Figure 8). A decrease in the number of bioactive peptides was observed in digested Ras cheese at 30 days of ripening with respect to the undigested corresponding sample, whereas for Ras cheese at 90 and 180 days of ripening, in vitro digestion brought about an increase in the number of identified bioactive peptides. In all the samples, the majority of bioactive peptides were derived from β-casein hydrolysis. The identified bioactive peptides presented various functionality such as ACE-inhibitory, DPP-IV-inhibitory, anti-microbial, anti-inflammatory, anti-cancer, anxiolytic, immunomodulatory and opioid activities. Finally, some phosphorylated peptides have been previously characterised as able to promote calcium uptake. The most frequent bioactivity was ACE-inhibition (81 peptides) followed by antioxidant activity (32 peptides), anti-microbial activity (19 peptides), DPP-IV-inhibition (18 peptides) and opioid activity (7 peptides). Some bioactive peptides were multi-functional and able to exert more than one activity.

#### 3.4.1. Evolution of ACE-Inhibitory Peptides during Ras Ripening and Digestion

**Figure 9:**
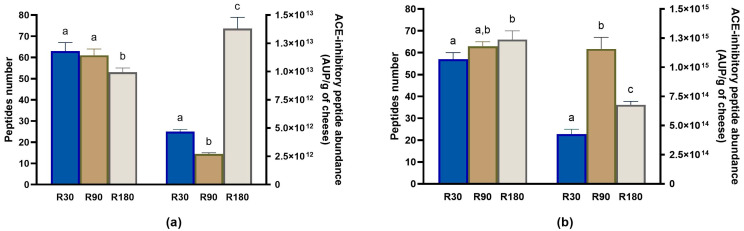
Peptidomics analysis of ACE-inhibitory peptides identified in undigested and in vitro digested Ras cheese at different ripening times. Analysis was carried out on low-molecular weight peptide fractions extracted by ultrafiltration at 3 kDa from undigested and in vitro digested Ras cheese samples at 30 (R30 sample), 90 (R90 sample) and 180 (R180 sample) days of ripening. (**a**) Number (**left** y-axis) and total abundance (**right** y-axis) of ACE-inhibitory peptides in undigested Ras cheese sample. (**b**) Number (**left** y-axis) and total abundance (**right** y-axis) of ACE-inhibitory peptides in in vitro digested Ras cheese sample. Abundance data are reported as the sum of the intensity of each identified ACE-inhibitory peptide measured as area under the peak (AUP) by Skyline analysis. The complete list of identified ACE-inhibitory peptides can be found in Supplementary Table S3, whereas the semi-quantitative data in Supplementary Table S4. Different letters among samples in the same assay denote significant differences (*p* < 0.05).

As reported above, 81 peptides displayed ACE-inhibitory activity. In cheese samples, the highest number of ACE-inhibitory peptides was found for Ras cheese at 30 days of ripening; then, their amount declined as the ripening time increased (Figure 9a). Vice versa, in digested samples, the number of ACE-inhibitory peptides increased accordingly to the ripening time, and the highest number was found for digested Ras cheese at 180 days of ripening (Figure 9b).

Semi-quantitative analysis revealed that the total ACE-inhibitory peptide abundance slightly decreased passing from 30 to 90 days of ripening. However, an evident increase in the total ACE-inhibitory peptide abundance occurred after 180 days of ripening (Figure 9a). Therefore, although the 180 day-ripened Ras sample contained the lowest number of ACE-inhibitory peptides, they were present in a higher concentration with respect to the Ras samples collected after 30 and 90 days of ripening. A similar trend in ACE-inhibitory peptide intensity as a function of the ripening time was already described for Parmigiano Reggiano and Asiago cheeses [7,36]. 

Considering the individual ACE-inhibitory peptides, semi-quantitative analysis revealed that some peptides were degraded, being present in the highest amount in Ras cheese at 30 days of ripening, whereas others, released during ripening, reached the maximum amount after 180 days of ripening (Supplementary Table S4). It is worth mentioning that the majority of ACE-inhibitory peptides displayed an increasing trend during ripening. In the case of ACE-inhibitory peptides that showed a decreasing trend during ripening, most of them derived from the regions 132–143 and 169–209 of β-casein. These regions contained preferred cleavage sites for lactic acid bacteria proteinases [9,40,41,42]. ACE-inhibitory peptides in these regions were easily released at early ripening stage and further hydrolysed into smaller peptides, some of which still presented ACE-inhibitory activity. For example, peptides NLHLPLPLL, NLHLPLP, LHLPLPL, LHLPLP, HLPLP and LPLPLL from the β-casein region 132–143 were released in a higher amount after 30 days of ripening, and their concentration started to decrease at 90 days of ripening and, for most of them, it dropped to zero after 180 days of ripening. Contemporaneously, the amount of the hydrolysis product LPLP increased by about 100 times from 30 to 180 days of ripening. 

After in vitro digestion, an increase in total ACE-inhibitory peptide abundance was observed in each sample (Figure 9b). Regarding the ripening time, the ACE-inhibitory peptide abundance reached the maximum value in digested Ras cheese at 90 days of ripening. Considering the individual peptides, the majority of them peaked in in vitro digested Ras cheese at 90 and 180 days of ripening. Overall, 12 ACE-inhibitory peptides identified in undigested samples were not detected in any digested samples, whereas 13 ACE-inhibitory peptides were newly released during in vitro digestion and not found in undigested samples.

Among the ACE-inhibitory peptides identified in in vitro digested samples, 12 have been reported to exert an in vivo anti-hypertensive effect. For example, the tripeptides VPP and IPP, originally isolated from fermented milk, trigger a reduction in blood pressure in human hypertensive and pre-hypertensive subjects and in spontaneously hypertensive rats (SHR) when orally administered in the amount of 3 to 10 mg/day [43,44,45,46]. Additionally, the αS1-casein-derived peptides RYLGY and AYFYPEL as well as the β-casein-derived peptides LHLPLP, HLPLP and KVLPVPQ displayed a hypotensive effect in SHR by decreasing the systolic blood pressure of values comprised between 20 and 31.5 mmHg [47,48,49,50]. Some of these peptides have been already detected in in vitro digested cheeses of different varieties. For example, the tripeptides IPP and VPP were identified and, somewhat quantified, in Parmigiano Reggiano, Gorgonzola and Cheddar cheeses after in vitro gastro-intestinal digestion [7,16,51], whereas the peptide HLPLP was detected after the in vitro digestion of several cheeses including Grana Padano, Maasdam, Gorgonzola, Cheddar and Parmigiano Reggiano [7,16,51]. Moreover, three peptides with an in vivo anti-hypertensive effect in SHR (AYFYPEL, YPFPGPIPN and VRGPFPIVV) were found in human plasma after milk consumption by healthy volunteers, suggesting that these peptides can be absorbed intact and may also exert their effect in humans [52].

#### 3.4.2. Evolution of Antioxidant Peptides during Ras Ripening and Digestion

**Figure 10:**
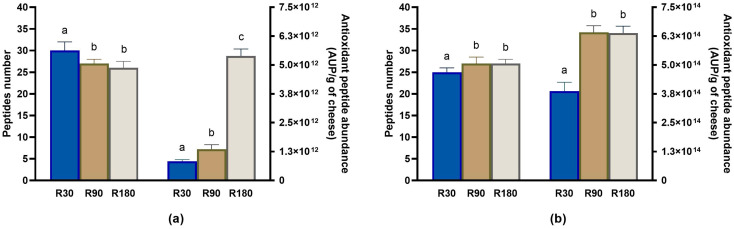
Peptidomics analysis of antioxidant peptides identified in undigested and in vitro digested Ras cheese at different ripening times. Analysis was carried out on low-molecular weight peptide fractions extracted by ultrafiltration at 3 kDa from undigested and in vitro digested Ras cheese samples at 30 (R30 sample), 90 (R90 sample) and 180 (R180 sample) days of ripening. (**a**) Number (**left** y-axis) and total abundance (**right** y-axis) of antioxidant peptides in undigested Ras cheese sample. (**b**) Number (**left** y-axis) and total abundance (**right** y-axis) of antioxidant peptides in in vitro digested Ras cheese sample. Abundance data are reported as the sum of the intensity of each identified antioxidant peptide measured as area under the peak (AUP) by Skyline analysis. The complete list of identified antioxidant peptides can be found in Supplementary Table S3, whereas the semi-quantitative data can be found in Supplementary Table S4. Different letters among samples in the same assay denote significant differences (*p* < 0.05).

The second most representative class of bioactive peptides by number identified in Ras cheeses before and after in vitro digestion was represented by antioxidant peptides. The number of identified antioxidant peptides decreased as the ripening time increased, and the highest number was found in Ras cheese at 30 days of ripening (Figure 10a). However, after in vitro digestion, the highest number of antioxidant peptides was found in digested Ras cheese at 90 and 180 days of ripening (Figure 10b).

Although the antioxidant peptides decreased in number during ripening, their total abundance increased passing from the Ras cheese at 30 days of ripening to Ras cheese at 90 days of ripening and above all to Ras cheese at 180 days of ripening and further increased after in vitro digestion in all the cheese samples, reaching the maximum values in digested Ras cheese at 90 and 180 days of ripening (Figure 10 and Supplementary Table S4). More than the 70% of identified antioxidant peptides (23 out of 32) contained in their sequence at least one Y residue, which is a feature considered of paramount importance in determining the antioxidant potential of a peptide [53]. Regardless of their bioavailability, antioxidant peptides can exert their biological activity in the gastro-intestinal tract, protecting it from oxidative damages caused by free radicals [53]. Nonetheless, some of the antioxidant peptides identified after the digestion of Ras cheese (such as AYFYPEL, YPFPGPIPN, VYPFPGPIPN, VLPVPQK, FPKYPVEPF and YQEPVLGPVR) were observed in human plasma after milk consumption [52].

#### 3.4.3. Evolution of DPP-IV-Inhibitory Peptides during Ras Ripening and Digestion

**Figure 11:**
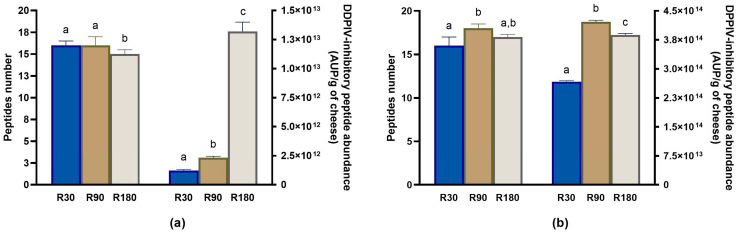
Peptidomics analysis of DPP-IV-inhibitory peptides identified in undigested and in vitro digested Ras cheese at different ripening times. Analysis was carried out on low-molecular weight peptide fractions extracted by ultrafiltration at 3 kDa from undigested and in vitro digested Ras cheese samples at 30 (R30 sample), 90 (R90 sample) and 180 (R180 sample) days of ripening. (**a**) Number (**left** y-axis) and total abundance (**right** y-axis) of DPP-IV-inhibitory peptides in undigested Ras cheese sample. (**b**) Number (**left** y-axis) and total abundance (**right** y-axis) of DPP-IV-inhibitory peptides in in vitro digested Ras cheese sample. Abundance data are reported as the sum of the intensity of each identified DPP-IV-inhibitory peptide measured as area under the peak (AUP) by Skyline analysis. The complete list of identified DPP-IV-inhibitory peptides can be found in Supplementary Table S3, whereas the semi-quantitative data can be found in Supplementary Table S4. Different letters among samples in the same assay denote significant differences (*p* < 0.05).

A total of 18 peptides with previously demonstrated DPP-IV-inhibitory activity were identified in undigested and digested Ras samples. The number of DPP-IV-inhibitory peptides did not differ so much between the different samples, and the majority of them was detected in all the undigested and digested samples (Figure 11).

However, the total DPP-IV-inhibitory peptide abundance displayed an increasing trend during ripening (Figure 11a and Supplementary Table S4). The relative amount of DPP-IV-inhibitory peptides almost doubled, passing from Ras cheese at 30 days of ripening to Ras cheese at 90 days of ripening and further increased by about 5.7 times after 180 days of ripening. Furthermore, in vitro digestion brought about an additional increase in total DPP-IV-inhibitory peptide abundance with the maximum value recorded in in vitro digested Ras cheese at 90 days of ripening (Figure 11b and Supplementary Table S4).

The majority of individual DPP-IV-inhibitory peptides displayed an increasing trend in their abundance during ripening, reaching the maximum amount in Ras cheese at 180 days of ripening (Supplementary Table S4). In in vitro digested samples, the highest intensity peak for individual DPP-IV-inhibitory peptides was found in digested Ras cheese at 90 and 180 days of ripening, with the only exception of the peptides SLPQNIPPL and VPYPQ, which exhibited a decreasing trend in their amount in the digested samples as a function of ripening.

Some of the DPP-IV-inhibitory peptides identified in in vitro digested samples (VPGEIVE, YPFPGPIPN, FPGPIPN, IPPLTQT, YPVEPF, LPLPL, LPLPLL and LPVPQ) have been previously found in the jejunum of healthy human volunteers after consuming a casein-rich meal [48]. In particular, the β-casein-derived peptide LPVPQ displayed an IC_50_ value against DPP-IV of about 40 μmol/L and could exert its inhibitory effect in vivo at intestinal level.

#### 3.4.4. Evolution of Other Bioactive Peptides during Ras Ripening and Digestion

Additional bioactive peptides detected quite frequently were anti-microbial and opioid peptides.

**Figure 12:**
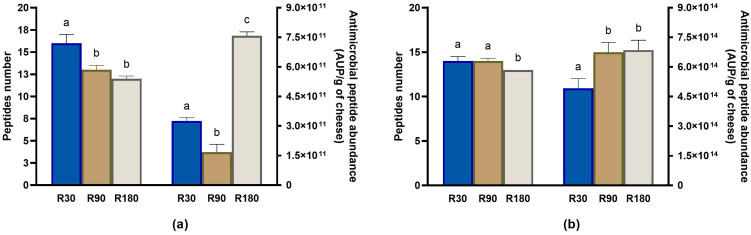
Peptidomics analysis of anti-microbial peptides identified in undigested and in vitro digested Ras cheese at different ripening times. Analysis was carried out on low-molecular weight peptide fractions extracted by ultrafiltration at 3 kDa from undigested and in vitro digested Ras cheese samples at 30 (R30 sample), 90 (R90 sample) and 180 (R180 sample) days of ripening. (**a**) Number (**left** y-axis) and total abundance (**right** y-axis) of anti-microbial peptides in undigested Ras cheese sample. (**b**) Number (**left** y-axis) and total abundance (**right** y-axis) of anti-microbial peptides in in vitro digested Ras cheese sample. Abundance data are reported as the sum of the intensity of each identified anti-microbial peptide measured as area under the peak (AUP) by Skyline analysis. The complete list of identified anti-microbial peptides can be found in Supplementary Table S3, whereas the semi-quantitative data can be found in Supplementary Table S4. Different letters among samples in the same assay denote significant differences (*p* < 0.05).

The highest number of anti-microbial peptides was found in Ras cheese at 30 days of ripening, and their number decreased during ripening (Figure 12a). However, the total anti-microbial peptide abundance was found to be maximum at the end of the ripening (Figure 12a and Supplementary Table S4).

In vitro digestion brought about an increase of more than one thousand times in anti-microbial peptides abundance (Figure 12b and Supplementary Table S4). The peptide intensity after in vitro digestion increased as a function of ripening, reaching a plateau after 90 days. Most of the β-casein anti-microbial peptides released after in vitro digestion have been also identified in vivo in the human gastro-intestinal tract where they may act modulating the gut microbiota composition [54,55]. For example, the β-casein-derived peptide EMPFPK was detected in an amount near to 1 mmol/L in human jejunum and was found to be able to inhibit the growth of pathogenic bacteria (such as *Escherichia coli* and *Streptococcus aureus*) but not of the beneficial lactobacilli [54,56]. Moreover, peptides released during the ripening and digestion of some aged cheeses may promote the growth of bifidobacteria and lactobacilli in vitro in faecal culture [57,58]. 

**Figure 13:**
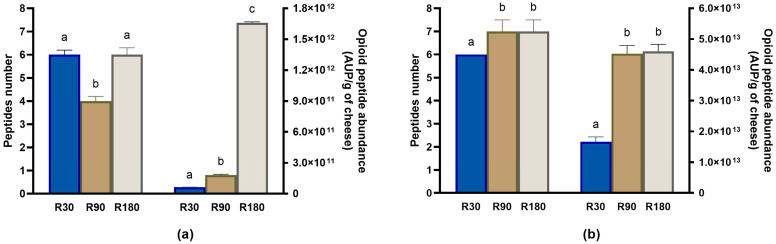
Peptidomics analysis of opioid peptides identified in undigested and in vitro digested Ras cheese at different ripening times. Analysis was carried out on low-molecular weight peptide fractions extracted by ultrafiltration at 3 kDa from undigested and in vitro digested Ras cheese samples at 30 (R30 sample), 90 (R90 sample) and 180 (R180 sample) days of ripening. (**a**) Number (**left** y-axis) and total abundance (**right** y-axis) of opioid peptides in undigested Ras cheese sample. (**b**) Number (**left** y-axis) and total abundance (**right** y-axis) of opioid peptides in in vitro digested Ras cheese sample. Abundance data are reported as the sum of the intensity of each identified opioid peptide measured as area under the peak (AUP) by Skyline analysis. The complete list of identified opioid peptides can be found in Supplementary Table S3, whereas the semi-quantitative data in Supplementary Table S4. Different letters among samples in the same assay denote significant differences (*p* < 0.05).

Concerning the bioactive peptides with opioid activity, their number did not differ so much among the digested and undigested samples, with the only exception being Ras cheese at 90 days of ripening that showed the lowest number of opioid peptides (Figure 13).

Nevertheless, the total opioid peptide abundance increased during ripening, and their amount almost tripled passing from 30 to 90 days of ripening and further increased by just over nine times from 90 to 180 days of ripening (Figure 13a and Supplementary Table S4). In vitro digestion caused a strong and ripening-dependent increase in the total opioid peptide abundance (Figure 13b and Supplementary Table S4). The highest peptide abundance was found in in vitro digested Ras cheese at 90 and 180 days of ripening. The impact of opioid peptides on human health is still a matter of debate. The most studied opioid peptide from milk is the peptide YPFPGPI (the so-called β-casomorphin-7), which exerts its activity through binding with the μ-opioid receptors in the gut and other organs. The binding of β-casomorphin-7 to its receptor in the gut was associated to an increased gastro-intestinal transit time and inflammation as well as an impairment in the gut barrier integrity [59,60]. In addition, some studies pointed out further negative effects of β-casomorphin-7 on human health, including type-2 diabetes and cardiovascular diseases [61]. However, other studies suggested positive health effects of opioid peptides on human health and in particular on the nervous system [62,63].

### 3.5. Quantification of the Bioactive Peptides VPP, IPP and APFPE in Ras Cheese Samples before and after In Vitro Digestion

The two anti-hypertensive tripeptides VPP and IPP as well as the potent DPP-IV-inhibitory peptide APFPE were quantified in the LMWPF extracted from undigested and in vitro digested Ras cheese samples. The results displayed in Table 1 highlighted that the concentration of the three quantified bioactive peptides increased during ripening reaching the maximum value after 180 days of ripening. 

Previous studies suggested that VPP and IPP concentration in cheese increased during ripening [7,64,65]. However, excessive ripening (i.e., more than 18/24 months) resulted in a decrease in VPP and IPP amount [7,64,65].

The concentration of IPP and VPP in in vitro digested Ras cheese at 30 and 90 days of ripening was significantly higher than that in the respective undigested samples, suggesting a continuous release during digestion. Vice versa, in in vitro digested Ras cheese at 180 days of ripening, the amount of IPP and VPP was not significantly different from that observed in undigested sample and significantly lower with respect to that recorded in the digested sample at 90 days of ripening. A similar behaviour was observed in in vitro digested Parmigiano Reggiano cheese as a function of the ripening time [7]. On the contrary, the concentration of APFPE continuously increased after digestion, depending on the ripening time, with the highest amount found in digested Ras cheese at 180 days of ripening. However, the amount of APFPE in this last sample was lower than that detected in the corresponding undigested sample.

Previous in vivo human studies have suggested that the peptides VPP and IPP were able to decrease blood pressure at doses comprised between 3 and 10 mg/day [43,44,45,46]. Therefore, it is possible to speculate that on the basis of the amount of VPP and IPP released during digestion, the consumption of 50–100 g of Ras cheese (and in particular Ras cheese at 90 days of ripening) could result in an in vivo effect.

## 4. Conclusions

The present study expands previous knowledge regarding the effect of ripening and in vitro gastro-intestinal digestion on the bioactive peptides and biological activities of cheese. By applying peptidomics techniques, we were able to track from a qualitative and semi-quantitative point of view the evolution of the bioactive peptide profiles in Ras cheese. Peptidomics analysis unveiled that ripening was pivotal to improve the biological activity and the bioactive peptides profiles of digested Ras cheese samples. Our results do not allow the identification of the generic best ripening time required for the development of optimum biological activities in other cheese varieties after in vitro digestion. Due to the different technological processes, manufacturing steps and ripening conditions, the utmost ripening time to achieve the best biological activities after digestion should be determined for each cheese variety. However, the present study lays the foundations for the application of peptidomics for future studies directed toward this topic.

The regular intake of fermented dairy products with the diet has been suggested to be of great importance to maintain a healthy diet due to the content of several essential nutrients. The presence of bioactive peptides generated during ripening and in vitro digestion, as demonstrated in the present study, pointed out a possible impact of cheese consumption on human health, especially in the prevention of cardiovascular diseases and diabetes. However, the majority of the studies on the positive effect of bioactive peptides have been carried out in vitro, highlighting the necessity of testing these compounds for their activity in vivo.

## Figures and Tables

**Table 1:** Changes in concentration of selected bioactive peptides during ripening and in vitro digestion of Ras cheese at 30 (R30), 90 (R90) and 180 (R180) days of ripening. Results are reported as mg/kg of cheese.

	Undigested Ras Cheese			Digested Ras Cheese		
Sequence	R30	R90	R180	R30	R90	R180
IPP	2.67 ± 0.11 ^a^	20.91 ± 1.02 ^b^	26.77 ± 1.65 ^c^	6.89 ± 0.41 ^d^	36.50 ± 2.69 ^e^	24.76 ± 1.33 ^c^
VPP	0.74 ± 0.02 ^a^	8.90 ± 0.59 ^b^	13.13 ± 0.98 ^c^	2.47 ± 0.19 ^d^	17.44 ± 1.09 ^e^	14.69 ± 1.07 ^c^
APFPE	3.27 ± 0.26 ^a^	9.58 ± 0.77 ^b^	82.09 ± 5.56 ^c^	13.59 ± 1.20 ^d^	17.15 ± 1.54 ^d^	52.01 ± 3.81 ^e^

Different letters in the same row indicate significantly different values (*p* < 0.05).

## Data Availability

The data presented in this study are available on request from the corresponding author.

## Supplementary Materials

The following supporting information can be downloaded at: https://www.mdpi.com/article/10.3390/biology12070948/s1, Figure S1: Heat map of αS1-casein-derived peptides in undigested Ras samples; Figure S2: Heat map of β-casein-derived peptides in undigested Ras samples; Figure S3: Venn diagrams showing differences between undigested and digested Ras samples; Table S1: Gros chemical composition (% *w*/*w*) of Ras cheese at different ripening times; Table S2: Skyline output with the mass spectrometry and semi-quantitative data of all the identified peptides; Table S3: List of identified bioactive peptides; Table S4: Semi-quantitative data of bioactive peptides.
