# Supplementary material for: Effect of Ripening and In Vitro Digestion on Bioactive Peptides Profile in Ras Cheese and Their Biological Activities
Source: Biology (Basel). 2023 Jul 2;12(7):948. doi: 10.3390/biology12070948 (PMC10376354; doi:10.3390/biology12070948)
Supplement: Supplementary file 1 [file biology-12-00948-s001.zip › Supplementary Figure captions.pdf]

Supplementary Figure captions.

Figure S1. Heat map showing differences in semi-quantitative data among αS1-casein individual peptides in undigested Ras cheese at different ripening times.

Figure S2. Heat map showing differences in semi-quantitative data among β-casein individual peptides in undigested Ras cheese at different ripening times.

Figure S3. Venn diagrams showing differences and similarity on the peptide profiles between undigested (Ras samples) and *in vitro* digested (D-Ras samples). Ras cheese at different ripening times.
