# Supplementary material for: Effect of Ripening and In Vitro Digestion on Bioactive Peptides Profile in Ras Cheese and Their Biological Activities
Source: Biology (Basel). 2023 Jul 2;12(7):948. doi: 10.3390/biology12070948 (PMC10376354; doi:10.3390/biology12070948)
Supplement: Supplementary file 1 [file biology-12-00948-s001.zip › Table_S1.pdf]

Table S1. Gros chemical composition (% w/w) of Ras cheese at different ripening times.

Ripening time (Days)	Dry matter (DM)	Fat	Fat in Dry Matter (%)	Protein	Soluble protein	Soluble protein coefficient (%)	Salt
30	63.6 ± 2.4[a]	33.4 ± 1.6[a]	52.5 ± 2.8[a]	25.5 ± 1.2[a]	3.2 ± 0.1[c]	12.6 ± 1.2[b]	2.3 ± 0.1[c]
90	65.1 ± 2.1[a]	34.3 ± 2.0[a]	52.7 ± 1.3[a]	25.9 ± 1.7[a]	4.6 ± 0.2[b]	17.8 ± 1.3[a]	3.2 ± 0.2[b]
180	67.3 ± 2.4[a]	35.2 ± 1.1[a]	52.3 ± 2.1[a]	27.8 ± 1.2[a]	5.3 ± 0.2[a]	19.0 ± 1.1[a]	3.9 ± 0.2[a]
