# Supplementary material for: Effect of Ripening and In Vitro Digestion on Bioactive Peptides Profile in Ras Cheese and Their Biological Activities
Source: Biology (Basel). 2023 Jul 2;12(7):948. doi: 10.3390/biology12070948 (PMC10376354; doi:10.3390/biology12070948)
Supplement: Supplementary file 1 [file biology-12-00948-s001.zip › Table_S3.pdf]

Table S3. Peptides sharing 100% of sequence homology with previously reported bioactive peptides identified in the low-molecular weight peptide fractions of un-digested and *in vitro* digested Ras cheese samples at different ripening time.

Peptide sequence	*Protein fragment*	*Bioactivity*	*Sample*
RELEEL	β-casein (1-6)	Antioxidant	R30, R90, R180, DR30, DR90 and DR180
LNVPGEIVE	β-casein (6-14)	ACE-inhibitor (IC_{50}=300 μmol/L)	R30, R90, R180, DR30, DR90 and DR180
NVPGEIVESL	β-casein (7-16)	Antioxidant	R30, R90, R180, DR30, DR90 and DR180
VPGEIVE	β-casein (8-14)	DPP-IV-inhibitor (IC_{50}=225 μmol/L)	R30, R90, R180, DR30, DR90 and DR180
S(pospho)S(pospho)S(pospho)EESITRIN	β-casein (17-27)	Anti-microbial	R30
FQS(pospho)EEQQQTEDELQDK	β-casein (33-48)	Promote calcium uptake	R30, R90, R180, DR30, DR90 and DR180
FQS(pospho)EEQQQTEDELQDKIHPF	β-casein (33-52)	Promote calcium uptake	R30, R90, DR30, DR90 and DR180
DKIHP	β-casein (47-51)	ACE-inhibitor (IC_{50}=113 μmol/L)	R30, R90, R180, DR30, DR90 and DR180
DKIHPF	β-casein (47-52)	ACE-inhibitor (IC_{50}=257 μmol/L)	R30, R90, R180, DR30, DR90 and DR180
LVYPFPGPI	β-casein (58-66)	ACE-inhibitor (IC_{50}=180 μmol/L)	R30, R90, DR30, DR90 and DR180
LVYPFPGPIP	β-casein (58-67)	ACE-inhibitor (IC_{50}=89 μmol/L)	R30, R90, R180, DR30, DR90 and DR180
VYPFPGPIPN	β-casein (59-68)	ACE-inhibitor (IC_{50}=325 μmol/L), antioxidant	R30, R90, R180, DR30, DR90 and DR180
YPFP	β-casein (60-63)	Opioid, anti-cancer	R30, R90, R180, DR30, DR90 and DR180
YPFPGPI	β-casein (60-66)	ACE-inhibitor (IC_{50}=500 μmol/L), antioxidant, opioid, immunomodulator, anxiolytic, anti-cancer	R30, R180, DR30, DR90 and DR180
YPFPGPIP	β-casein (60-67)	ACE-inhibitor (IC_{50}=224 μmol/L), antioxidant	R30, R90, R180, DR30, DR90 and DR180
YPFPGPIPN	β-casein (60-68)	ACE-inhibitor (IC_{50}=15 μmol/L), DPP-IV-inhibitor (IC_{50}=670 μmol/L), antioxidant	R30, R90, R180, DR30, DR90 and DR180
PFP	β-casein (61-63) αS1-casein (27-29)	ACE-inhibitor (IC_{50}=144 μmol/L)	R30, R90, R180 and DR180
PFPGPIPN	β-casein (61-68)	ACE-inhibitor	R30, R90, R180, DR30, DR90 and DR180

FPGPIPN	β-casein (62-68)	DPP-IV-inhibitor (IC_{50}=260 μmol/L)	R30, R90, R180, DR30, DR90 and DR180
PGPIPN	β-casein (63-68)	ACE-inhibitor, immunomodulator, anti-cancer, anti-inflammatory	R90, R180, DR30, DR90 and DR180
SLPQ	β-casein (69-72)	ACE-inhibitor (IC_{50}=330 μmol/L)	R30, R90, R180, DR30, DR90 and DR180
SLPQNIPPL	β-casein (69-77)	DPP-IV-inhibitor	R30, R90, R180, DR30, DR90 and DR180
NIPPLTQTPV	β-casein (73-82)	ACE-inhibitor (IC_{50}=173 μmol/L)	R30, R90, DR30, DR90 and DR180
NIPPLTQTPVVVPPFLQ	β-casein (73-89)	ACE-inhibitor (IC_{50}=450 μmol/L)	DR30, DR90 and DR180
IPP	β-casein (74-76)<br>κ-casein (108-110)	ACE-inhibitor (IC_{50}=5 μmol/L), DPP-IV-inhibitor (IC_{50}=169 μmol/L), antioxidant, anti-inflammatory	R30, R90, R180, DR30, DR90 and DR180
IPPLTQT	β-casein (74-80)	DPP-IV-inhibitor (IC_{50}=465 μmol/L)	R90 and DR90
TQTPVVVPPFLQPE	β-casein (78-91)	Antioxidant	R30, R90, R180, DR30, DR90 and DR180
TPVVVPPFLQP	β-casein (80-90)	ACE-inhibitor (IC_{50}=749 μmol/L)	R30, R90, R180, DR30, DR90 and DR180
VVPP	β-casein (83-86)	ACE-inhibitor (IC_{50}=258 μmol/L)	R30, R90, R180, DR30, DR90 and DR180
VPP	β-casein (84-86)	ACE-inhibitor (IC_{50}=8 μmol/L), antioxidant, anti-inflammatory	R30, R90, R180, DR90 and DR180
EAMAPK	β-casein (100-105)	Anti-microbial	R30, R90, R180, DR30, DR90 and DR180
HKEMPFPK	β-casein (106-113)	Anti-microbial	R90, R180, DR30, DR90 and DR180
EMPFPK	β-casein (108-113)	Anti-microbial	R30, R90, R180, DR30, DR90 and DR180
FPKYPVEPF	β-casein (111-119)	Antioxidant	R30, R90, R180 and DR90
KYP	β-casein (113-115)	ACE-inhibitor (IC_{50}=364 μmol/L)	R30, R90 and R180
YPVEPF	β-casein (114-119)	DPP-IV-inhibitor (IC_{50}=125 μmol/L), antioxidant, anti-microbial, opioid	R30, R90, R180, DR30, DR90 and DR180
DVENLHLPLPL	β-casein (129-139)	Anti-microbial	R30, DR30, DR90 and DR180
NLHLPLPLL	β-casein (132-140)	ACE-inhibitor (IC_{50}=15 μmol/L)	R30, R90, DR90 and DR180
NLHLPLP	β-casein (132-138)	ACE-inhibitor (IC_{50}=51 μmol/L)	R30, R90, DR30, DR90 and DR180
LHLPLPL	β-casein (133-139)	ACE-inhibitor (IC_{50}=425 μmol/L)	R30, DR30, DR90 and DR180
LHLPLP	β-casein (133-138)	ACE-inhibitor (IC_{50}=3 μmol/L)	R30, DR90 and DR180

HLPLP	β-casein (134-138)	ACE-inhibitor (IC_{50}=22 μmol/L)	R30, R90, DR30, DR90 and DR180
LPLPLL	β-casein (135-140)	ACE-inhibitor, DPP/IV-inhibitor (IC_{50}=372 μmol/L)	R30, R90, R180, DR30, DR90 and DR180
LPLPL	β-casein (135-139)	DPP/IV-inhibitor (IC_{50}=325 μmol/L)	R30, R90, R180, DR30, DR90 and DR180
LPLP	β-casein (135-138)	ACE-inhibitor (IC_{50}=750 μmol/L)	R30, R90, R180, DR30, DR90 and DR180
LQSW	β-casein (140-143)	ACE-inhibitor (IC_{50}=500 μmol/L)	R30, R90, R180, DR30, DR90 and DR180
WMHQPHQPLPPT	β-casein (143-154)	ACE-inhibitor, anti-inflammatory	DR30, DR90 and DR180
HQPHQPLPPT	β-casein (145-154)	ACE-inhibitor	R30, R90, DR30, DR90 and DR180
SKVLPVPQ	β-casein (168-175)	ACE-inhibitor (IC_{50}=39 μmol/L)	R90, DR30, DR90 and DR180
KVLPVPQ	β-casein (169-175)	ACE-inhibitor (IC_{50}=1000 μmol/L), anti-inflammatory	R30, R90, R180, DR30, DR90 and DR180
VLPVPQK	β-casein (169-175)	ACE-inhibitor (IC_{50}=15 μmol/L), antioxidant, anti-microbial	R30, R90, DR30, DR90 and DR180
LPVPQ	β-casein (171-175)	DPP-IV-inhibitor (IC_{50}=44 μmol/L)	R30, R90, R180, DR30, DR90 and DR180
VPYPQR	β-casein (178-183)	Antioxidant	R30, R90 and R180
VPYPQ	β-casein (178-182)	DPP-IV-inhibitor (IC_{50}=41 μmol/L), antioxidant	R30, R90, R180, DR30, DR90 and DR180
PYPQ	β-casein (179-182)	Antioxidant	R30, R90, R180, DR30, DR90 and DR180
RDMPIQAF	β-casein (183-190)	ACE-inhibitor (IC_{50}=209 μmol/L)	R30 and R90
LLY	β-casein (191-193)	Antioxidant, immunomodulator, anti-inflammatory	R30, R90, DR30, DR90 and DR180
LYQEPVLGPVR	β-casein (192-203)	ACE-inhibitor, anti-inflammatory	DR30, DR90 and DR180
YQEPVLGPVR	β-casein (193-203)	ACE-inhibitor (IC_{50}=3300 μmol/L), antioxidant, immunomodulator, anti-inflammatory	R30, R90, R180, DR30, DR90 and DR180
YQEP	β-casein (193-196)	ACE-inhibitor (IC_{50}=317 μmol/L), antioxidant	R30, R90 and R180
YQEPVLGPVRGPFPI	β-casein (193-207)	Anti-microbial	R30, R90, R180, DR30, DR90 and DR180
YQEPVLGPVRGPFPIIV	β-casein (193-209)	ACE-inhibitor (IC_{50}=101 μmol/L), anti-microbial, immunomodulator	R30, R90, R180, DR30, DR90 and DR180
QEPV	β-casein (194-197)	Immunomodulator	R30, R180, DR30, DR90 and DR180

QEPVLGPVRGPFPIIV	β-casein (194-209)	ACE-inhibitor (IC_{50}=600 μmol/L)	R30, R90, DR30, DR90 and DR180
EPVLGPVRGPFP	β-casein (195-206)	ACE-inhibitor (IC_{50}=790 μmol/L)	R30, R90, R180, DR30, DR90 and DR180
VLGPVRGPFP	β-casein (197-206)	ACE-inhibitor (IC_{50}=137 μmol/L)	R30, R90 and R180
VRGPFPIIV	β-casein (201-209)	ACE-inhibitor (IC_{50}=600 μmol/L)	R30, R90, R180, DR90 and DR180
VRGPFP	β-casein (201-206)	ACE-inhibitor (IC_{50}=592 μmol/L)	R30, R90 and R180
RPK	αS1-casein (1-3)	ACE-inhibitor (IC_{50}=92 μmol/L), anti-cancer	DR30, DR90 and DR180
RPKHPI	αS1-casein (1-6)	ACE-inhibitor (IC_{50}=40 μmol/L)	R30, R90, R180, DR30 and DR90
RPKHPIK	αS1-casein (1-7)	Anti-microbial	R30 and R180
VLNENLLR	αS1-casein (15-22)	Anti-microbial	R30, R90 and R180
ENLLRF	αS1-casein (18-23)	ACE-inhibitor (IC_{50}=82 μmol/L),	R30, R90, R180, DR90 and DR180
LRFF	αS1-casein (21-24)	ACE-inhibitor (IC_{50}=117 μmol/L),	R30 and DR180
FVAPFPEVFG	αS1-casein (24-33)	ACE-inhibitor (IC_{50}=650 μmol/L)	R30, R90, R180, DR30, DR90 and DR180
APFPE	αS1-casein (26-30)	DPP-IV-inhibitor (IC_{50}=49 μmol/L)	R30, R90, R180, DR30, DR90 and DR180
PFPEVFGK	αS1-casein (27-34)	ACE-inhibitor (IC_{50}=108 μmol/L)	R30, R90 and R180
FPEVFGK	αS1-casein (28-34)	ACE-inhibitor (IC_{50}=140 μmol/L)	R30, R90 and R180
DIGS(pospho)ES(pospho)TEDQAMEDIK	αS1-casein (43-58)	Promote calcium uptake	R90, R180, DR30, DR90 and DR180
VPSERY	αS1-casein (86-91)	ACE-inhibitor (IC_{50}=706 μmol/L)	R30, R90 and R180
VPSERYL	αS1-casein (86-92)	ACE-inhibitor (IC_{50}=250 μmol/L)	R30, R90 and R180
RYLGY	αS1-casein (90-94)	ACE-inhibitor (IC_{50}=3 μmol/L), antioxidant	R30 and DR30
YLG	αS1-casein (91-93)	Antioxidant	R30, R90, R180, DR30, DR90 and DR180
YLGY	αS1-casein (91-94)	Antioxidant	R30, R90, R180, DR30 and DR180
YLGYLE	αS1-casein (91-96)	ACE-inhibitor (IC_{50}=86 μmol/L), antioxidant, opioid	R30, R90, R180, DR90 and DR180
YLGYLEQ	αS1-casein (91-97)	Anxiolytic	R30, R90 and R180
LGY	αS1-casein (92-94)	ACE-inhibitor (IC_{50}=22 μmol/L), antioxidant	R30, R90, R180, DR30, DR90 and DR180
YLEQLLR	αS1-casein (94-100)	Anti-microbial	R30, R90, R180, DR30 and DR90
YKVPQL	αS1-casein (104-109)	ACE-inhibitor (IC_{50}=22 μmol/L)	R30, R90, R180, DR90 and DR180
LHSMK	αS1-casein (104-109)	Antioxidant	DR30, DR90 and DR180

AYFYPE	αS1-casein (143-148)	ACE-inhibitor (IC_{50}=22 μmol/L), antioxidant	R30, R90, R180, DR30, DR90 and DR180
AYFYPEL	αS1-casein (143-149)	ACE-inhibitor (IC_{50}=7 μmol/L), antioxidant, opioid	R30, R180, DR30, DR90 and DR180
YFYPE	αS1-casein (144-148)	Opioid	R30, R90, R180, DR30, DR90 and DR180
YFYPEL	αS1-casein (144-149)	ACE-inhibitor (IC_{50}=9 μmol/L), antioxidant, opioid	DR30, DR90 and DR180
PEL	αS1-casein (147-149)	Antioxidant	R30, R90, R180, DR30, DR90 and DR180
DAYPSGAW	αS1-casein (157-164)	ACE-inhibitor (IC_{50}=98 μmol/L)	R90, R180, DR30, DR90 and DR180
GAW	αS1-casein (162-164)	ACE-inhibitor (IC_{50}=240 μmol/L)	DR30, DR90 and DR180
SDIPNPIGSENSEK	αS1-casein (147-149)	Anti-microbial	R30, R90, R180, DR30, DR90 and DR180
NMAINPSK	αS2-casein (25-32)	ACE-inhibitor (IC_{50}=600 μmol/L)	DR30, DR90 and DR180
KALNEINQF	αS2-casein (80-88)	Antioxidant	R30, R90, DR90 and DR180
YQKFPQY	αS2-casein (89-95)	ACE-inhibitor (IC_{50}=98 μmol/L), antioxidant	R30, R90 and R180
NPWDQ	αS2-casein (107-111)	Immunomodulator	R30, R90, R180, DR30, DR90 and DR180
VPITPT	αS2-casein (117-122)	DPP-IV-inhibitor (IC_{50}=130 μmol/L)	R30, R90, R180, DR30, DR90 and DR180
LKKISQ	αS2-casein (164-169)	Anti-microbial	R30
FALPQY	αS2-casein (174-179)	ACE-inhibitor (IC_{50}=4 μmol/L)	R30, R90 and R180
AMKPW	αS2-casein (189-193)	ACE-inhibitor (IC_{50}=580 μmol/L)	R30, R90, R180, DR30, DR90 and DR180
WIQP	αS2-casein (193-196)	ACE-inhibitor (IC_{50}=14 μmol/L), DPP-IV-inhibitor (IC_{50}=237 μmol/L)	R30, R90, R180, DR90 and DR180
TKVIP	αS2-casein (198-202)	ACE-inhibitor (IC_{50}=400 μmol/L)	R30, R90, R180, DR30, DR90 and DR180
TKVIPYVRYL	αS2-casein (193-207)	Anti-microbial	R30, R90 and R180
EKDERF	κ-casein (12-17)	ACE-inhibitor (IC_{50}=14 μmol/L)	R30, R90 and R180
DERF	κ-casein (14-17)	ACE-inhibitor (IC_{50}=38 μmol/L)	R30, R90, R180, DR30, DR90 and DR180
FFSDK	κ-casein (17-21)	Anti-cancer, anti-microbial	R30, R90, DR30, DR90 and DR180
IAK	κ-casein (22-24)	ACE-inhibitor (IC_{50}=16 μmol/L)	DR30, DR90 and DR180

IPIQY	κ-casein (26-30)	DPP-IV-inhibitor (IC_{50}=35 μmol/L)	R30, R90, R180, DR30, DR90 and DR180
YVL	κ-casein (30-32)	Antioxidant, anti-microbial	R30, R90, R180, DR30, DR90 and DR180
YGL	κ-casein (38-40)	ACE-inhibitor (IC_{50}=409 μmol/L)	R30, R90, R180, DR30 and DR180
INNQFLPYPY	κ-casein (51-60)	DPP-IV-inhibitor (IC_{50}=40 μmol/L)	R30, DR30, DR90 and DR180
LPYPY	κ-casein (56-60)	ACE-inhibitor (IC_{50}=29 μmol/L), DPP-IV-inhibitor (IC_{50}=108 μmol/L)	R30, R90, R180, DR30, DR90 and DR180
IIAEK	β-lactoglobulin (71-75)	ACE-inhibitor (IC_{50}=64 μmol/L)	DR30, DR90 and DR180
IAEK	β-lactoglobulin (72-75)	ACE-inhibitor (IC_{50}=26 μmol/L)	DR30, DR90 and DR180
VLDTDYK	β-lactoglobulin (94-100)	ACE-inhibitor (IC_{50}=946 μmol/L)	DR90 and DR180
YLL	β-lactoglobulin (78-80)	ACE-inhibitor (IC_{50}=519 μmol/L), antioxidant	R30, R90, R180, DR30, DR90 and DR180
TPEVDDEALEK	β-lactoglobulin (125-135)	DPP-IV-inhibitor (IC_{50}=320 μmol/L), anti-microbial	DR30, DR90 and DR180
ALPM	β-lactoglobulin (142-145)	ACE-inhibitor (IC_{50}=928 μmol/L)	DR30, DR90 and DR180
ALPMH	β-lactoglobulin (142-146)	ACE-inhibitor (IC_{50}=521 μmol/L)	DR30, DR90 and DR180

Peptide bioactivities and IC_{50} values were obtained from the Milk Bioactive Peptides Database.
Abbreviations are: R30: Ras cheese at 30 days of ripening; R90: Ras cheese at 90 days of ripening; R180: Ras cheese at 180 days of ripening; DR30: digested Ras cheese at 30 days of ripening; DR90: digested Ras cheese at 90 days of ripening; DR180: digested Ras cheese at 180 days of ripening; ACE: angiotensin-converting enzyme; DPP-IV: dipeptidyl peptidase IV; PEP: prolyl-endopeptidase.
