# Supplementary figures and images for: Effect of Ripening and In Vitro Digestion on Bioactive Peptides Profile in Ras Cheese and Their Biological Activities
Source: Biology (Basel). 2023 Jul 2;12(7):948. doi: 10.3390/biology12070948 (PMC10376354; doi:10.3390/biology12070948)

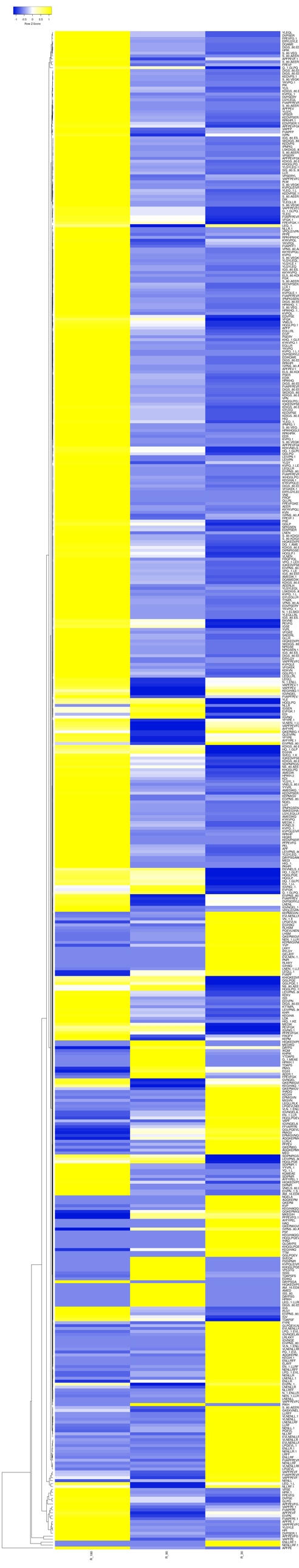

Supplement: Supplementary file 1 [file biology-12-00948-s001.zip › Figure_S1.png]

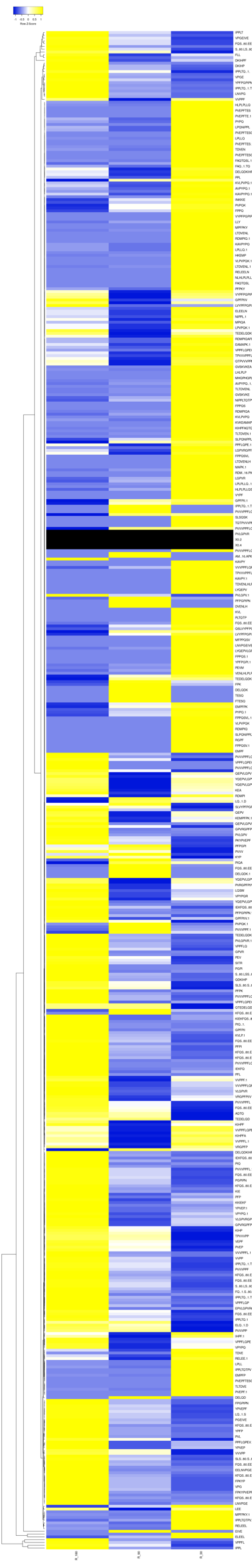

Supplement: Supplementary file 1 [file biology-12-00948-s001.zip › Figure_S2.png]
